# Break-in Period ≤24 Hours as an Option for Urgent-start Peritoneal Dialysis in Patients With Diabetes

**DOI:** 10.3389/fendo.2022.936573

**Published:** 2022-07-14

**Authors:** Xiaoqing Hu, Liming Yang, Zhanshan Sun, Xiaoxuan Zhang, Xueyan Zhu, Wenhua Zhou, Xi Wen, Shichen Liu, Wenpeng Cui

**Affiliations:** ^1^ Division of Nephrology, The Second Hospital of Jilin University, Changchun, China; ^2^ Division of Nephrology, The First Hospital of Jilin University-the Eastern Division, Changchun, China; ^3^ Division of Nephrology, Xing’anmeng people’s Hospital, Inner Mongolia, China; ^4^ Division of Nephrology, Jilin FAW General Hospital, Changchun, China; ^5^ Division of Nephrology, Jilin City Central Hospital, Jilin, China

**Keywords:** end-stage renal disease, urgent start peritoneal dialysis, diabetics, break-in period, complications

## Abstract

**Background:**

The optimal break-in period (BI) of urgent-start peritoneal dialysis (USPD) initiation for patients with end-stage renal disease (ESRD) and diabetes is unclear. We aimed to explore the safety and applicability of a BI ≤24 h in patients with ESRD and diabetes.

**Methods:**

We used a retrospective cohort design wherein we recruited patients with ESRD and diabetes who underwent USPD at five institutions in China between January 2013 and August 2020. The enrolled patients were grouped according to BI. The primary outcomes were mechanical and infectious complication occurrences, whereas the secondary outcome was technique survival.

**Results:**

We enrolled 310 patients with diabetes, of whom 155 and 155 patients were in the BI ≤24 h and BI >24 h groups, respectively. The two groups showed a comparable incidence of infectious and mechanical complications within 6 months after catheter insertion (*p*>0.05). Logistic regression analysis revealed that a BI ≤24 h was not an independent risk factor for mechanical or infectious complications. Kaplan–Meier estimates showed no statistically significant between-group differences in technique survival rates (*p*>0.05). Cox multivariate regression analysis revealed that a BI ≤24 h was not an independent risk factor for technique failure.

**Conclusion:**

USPD initiation with a BI ≤24 h may be safe and feasible for patients with ESRD and diabetes.

## Introduction

There is a global increase in the number of patients with end-stage renal disease (ESRD). Many of these patients require an urgent commencement of dialysis owing to late referral or an accidental deterioration of residual renal function (1, 2). Urgent-start hemodialysis (HD) *via* a central venous catheter is usually chosen in an unplanned dialysis method, but this technique could increase the prevalence of central venous stenosis, bacteremia, and thrombosis (3–5). In contrast, peritoneal dialysis (PD) has more potential benefits than HD, including cost-effectiveness, the preservation of residual renal function, and lifestyle flexibility. Urgent-start PD (USPD) is defined as the initiation of PD therapy within 2 weeks (6, 7) or 3 days after catheter insertion (8, 9). Most recent studies have demonstrated that USPD may be an adoptable dialysis option (1, 10, 11).

Several studies have reported complications related to USPD (1, 7, 10, 12–14). Some scholars argued that there may be an increased risk of dialysate leakage and catheter migration when dialysis is initiated urgently after catheter insertion (1, 12–14), whereas others hold the opposite view (7, 10). Patients undergoing PD have an increased intra-abdominal pressure due to the volume of dialysate infused into the peritoneal cavity, which can lead to anatomical complications in the abdominal wall. Patients with ESRD and diabetes are more susceptible to infections and poor wound healing due to high blood glucose levels (15, 16). We speculate that if PD is initiated urgently, patients with diabetes may be more likely to have mechanical and infectious complications than patients with adequate break-in periods (BIs). However, since there are no reports of a BI ≤24 h in patients with diabetes, the optimal BI for diabetic patients with ESRD is unclear.

Therefore, in this study, we compared dialysis-related complications, and PD technique survival rates between patients with BI ≤24 h and BI >24 h in a large sample population. The aim was to determine the safety and applicability of a BI ≤24 h as an urgent method of initiating dialysis in patients with diabetes.

## Methods

### Study Design and Patient Selection

This real-world study used a retrospective cohort design. The inclusion criteria were patients diagnosed with ESRD between January 2013 and August 2020 at five institutions (The Second Hospital of Jilin University, The First Hospital of Jilin University—the Eastern Division, Jilin City Center Hospital, Jilin FAM General Hospital, and Xing’anmeng People’s Hospital,). The indications for USPD were as follows: uremia symptoms (such as gastrointestinal symptoms and consciousness alteration), severe volume overload or pulmonary edema, hyperkalemia (K >6.5 mmol/L), and severe acidosis (serum bicarbonate < 10 mEq/L) as we described previously (17). Patients were excluded if they exhibited any of the following: 1) non-USPD, 2) incomplete data, 3) age younger than 18 years, 4) those who received chronic HD therapy before and/or after PD initiation, 5) percutaneous catheter placement and laparoscopic surgery, and 6) patients without diabetes.

### Catheter Implantation and Dialysis Prescription

Catheter implantation was performed in a standardized manner at each PD center. Double-cuffed Tenckhoff catheters were inserted under local anesthesia during an open surgery for all patients as described previously (18). First, a nephrologist made a left paramedian incision 9–13 cm above the pubic symphysis. Subcutaneous tissue was carefully detached to reach the anterior sheath of the rectus muscle, and a 2–4 cm incision was made over the anterior rectus sheath. Subsequently, the posterior sheath was incised, and the peritoneum was exposed using blunt dissection. Purse-string suturing was performed along the small opening in the peritoneum. The PD catheter was then inserted into the peritoneal cavity. The correct positioning of the catheter tip was tested by assessing patient sensations and the free flow of saline into and out of the abdominal cavity. Thereafter, the purse-string suture was tightened and tied. Finally, the catheter was pulled through the exit site *via* a subcutaneous tunnel (18). All clinicians performed the procedures had received specialized training in catheter implantation. The number of clinicians who performed PD catheter implantation was four, two, two, one and one in The Second Hospital of Jilin University, The First Hospital of Jilin University—the Eastern Division, Jilin City Center Hospital, Jilin FAM General Hospital, and Xing’anmeng People’s Hospital, respectively.

During the first few days of dialysis, the exchange volume for both groups was 0.5–1.0 L. In the absence of PD-related complications, such as dialysate leakage, the exchange volume was gradually increased to 2 L within 2 weeks. Continuous ambulatory PD or automated PD was available during the initiation period. Patients were adequately educated on PD, including dialysate exchange and catheter care. Patients were followed up every 3–6 months to monitor the adequacy of PD, including weekly measurement of Kt/V_urea_ and weekly creatinine clearance, with targets of ≥1.7 and >50 L/week/1.73 m^2^, respectively.

Primary outcomes were the occurrences of early mechanical and infectious complications. Complications were examined up to 6 months following PD catheter insertion. All patients with complications initially received conservative treatments. If the complications were not resolved, surgical interventions were performed with the patient’s informed consent. Mechanical complications included dialysate leakage, bleeding, catheter migration, and omental wrap. Infectious complications included peritonitis, exit-site infection, and tunnel infection. The secondary outcome was technique survival.

Catheter migration was defined as a drainage outflow volume significantly less than the inflow volume and the location of the catheter tip outside the true pelvis, which was confirmed by abdominal radiography (7). Dialysate leakage was defined as the loss of dialysate from the peritoneal cavity, or the appearance of dialysate at the exit site. Anatomical dialysate leakage to other areas was confirmed by visual observation, computed tomography, ultrasonography, or the methylene blue method. Bleeding episodes were defined as blood loss into dialysate that required hemostatic drugs, blood transfusion, or surgical intervention for hemostasis. Omental wrap was proven by secondary surgery. Technique failure was defined as conversion from PD to HD for at least 30 days (19, 20). Chronic HD was defined as an HD program lasting for >3 months and >7 sessions of HD monthly (21). Temporary HD was defined as HD treatment within 3 months before and/or after PD initiation.

### Data Collection

The following data were collected: 1) patients’ demographics, including sex, age, presence or absence of temporary HD, cause of ESRD, comorbidities, the history of abdominal surgery, date of PD initiation, and date of catheter insertion; 2) preoperative laboratory indicators, including the levels of white blood cells (WBC), hemoglobin (Hb), blood albumin (Alb), triglycerides (TG), total cholesterol (TC), high-density lipoprotein cholesterol (HDL), low-density lipoprotein cholesterol (LDL), blood creatinine (Cr), blood uric acid (UA), blood urea nitrogen (BUN), estimated glomerular filtration rate(eGFR), blood potassium (K), blood sodium (Na), blood calcium (Ca), blood phosphorus (P), and blood glucose (BG); and 3) complications and outcome events, including date(s) of mechanical and infectious complications, treatment and outcome of complications, and date of technique failure.

### Statistical Analysis

Statistical analyses were performed using SPSS Statistics version 25.0 (IBM Corp, Armonk, NY, USA). Measurement data were expressed as the mean ± standard deviation, and the *t*-test was used for between-group comparisons of normally distributed data; otherwise, the data were expressed as median (interquartile range), and the Wilcoxon rank sum test was used for between-group comparisons of non-normally distributed data. Comparisons between groups of count data were performed using the chi-square or Fisher exact test and expressed as numbers and percentages. Factors associated with complications were determined using logistic regression analysis. To avoid missing important risk factors in the multivariate logistic regression analysis, the *p*-value for significance was relaxed to 0.2. In statistical language, a *p*-value <0.2 is acceptable (22). Technique survival rates were assessed using the Kaplan–Meier method and the differences between the two groups were compared by the log-rank test. Factors associated with technique failure were determined using Cox multivariate regression analysis. Covariates with *p*-value < 0.2 in the univariate analysis were used for multivariate regression. Graphs were plotted using GraphPad Prism (GraphPad Software, Armonk, NY, USA). A *p*-value <0.05 was considered statistically significant. In this study, we performed consecutive sampling of patients in the five PD centers who met the eligibility criteria.

## Results

### Patients’ Characteristics

**Figure 1:**
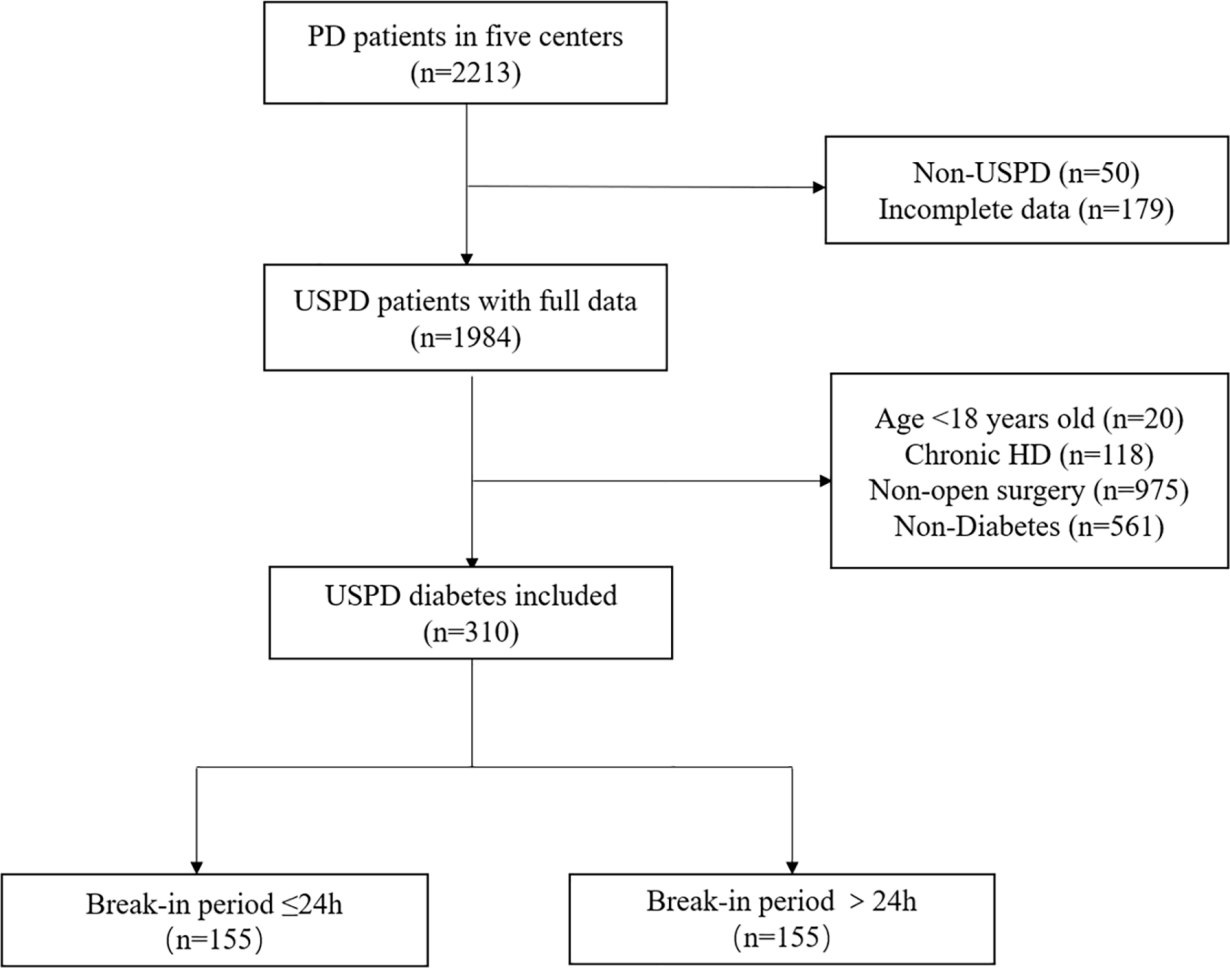
Flowchart. PD, peritoneal dialysis; USPD: urgent start PD; HD, hemodialysis.

As shown in **Figure 1**, this study included 310 patients with diabetes who underwent PD, including 155 patients in the BI ≤24 h group (50%) and 155 patients in the BI >24 h group (50%). Patient baseline characteristics are presented in **Table 1**. The mean age of patients was 56.56 years, and 202 (65.2%) patients were men. Compared to the BI >24 h group, the BI ≤24 h group had more men (71.0% versus 59.4%, p=0.032) and fewer cases of temporary HD (23.2% versus 41.3%, p=0.001) "should be "Compared to the BI >24 h group, the BI ≤24 h group had more men (71.0% versus 59.4%, p=0.032) , fewer cases of temporary HD (23.2% versus 41.3%, p=0.001) and shorter BIs(1 vs 4, p=0.000) however, there were no significant between-group differences in the other measured parameters.

**Table 1:** Baseline characteristics of patients in different BI groups.

	Overall (n=300)	BI ≤ 24 h (n=155)	BI > 24h (n=155)	x^2^/z/t-value	*p*-value
Sex (men %)	202 (65.2%)	110 (71.0%)	92 (59.4%)	4.604	0.032
Age (years)	56.56 ± 12.16	55.25 ± 12.65	57.88 ± 11.55	-1.908	0.057
Temporary HD [n (%)]	100 (32.3%)	36 (23.2%)	64 (41.3%)	11.573	0.001
Cause of ESRD [n (%)]				6.002	0.409
CGN	22 (7.1%)	9 (5.8%)	13 (8.4%)		
Diabetes	253 (81.6%)	127 (81.9%)	126 (81.3%)		
hypertension	16 (5.2%)	11 (7.1%)	5 (3.2%)		
Interstitial nephritis	2 (0.6%)	0 (0.00%)	2 (1.3%)		
PKD	1 (0.3%)	0 (0.00%)	1 (0.6%)		
Others	3 (1.0%)	1 (0.6%)	2 (1.3%)		
Unknown cause	13 (4.2%)	7 (4.5%)	6 (3.9%)		
Comorbidities [n (%)]					
Hypertension	303 (97.7%)	151 (97.4%)	152 (98.1%)	0.000	1.000
Abdominal surgery history [n (%)]	37 (11.9%)	13 (8.4%)	24 (15.5%)	3.713	0.054
Break-in period (d)	1.5 (1,4)	1 (0,1)	4 (3,5)	-15.51	0.000
Laboratory indicators					
WBC (10*9/L)	7.05 (5.50,8.60)	6.91 (5.56,8.60)	7.10 (5.50,8.80)	-0.824	0.410
Hb (g/l)	87.00 (76.00,101.00)	89.00 (76.00,104.00)	86.00 (76.00,99.00)	-0.856	0.392
Alb (g/L)	32.50 (29.28,36.35)	32.10 (28.90,36.00)	32.70 (29.63,36.90)	-1.369	0.171
TG (mmol/L)	1.56 (1.34,1.90)	1.56 (1.21,2.02)	1.56 (1.44,1.83)	-0.468	0.640
TC (mmol/L)	4.43 (3.97,4.97)	4.43 (3.72,5.02)	4.43 (4.26,4.81)	-1.010	0.312
HDL (mmol/L)	0.94 (0.86,1.07)	0.94 (0.83,1.11)	0.94 (0.92,1.06)	-0.170	0.865
LDL (mmol/L)	2.62 (2.29,2.97)	2.62 (2.20,2.96)	2.62 (2.40,3.00)	-0.980	0.327
Cr (µmol/L)	650.00 (521.74,842.30)	647.00 (532.20,801.00)	666.70 (511.20,885.90)	-0.715	0.474
UA (µmol/L)	417.00 (340.75,498.08)	418.00 (348.00,505.00)	417.00 (327.00,484.00)	-1.565	0.118
BUN (mmol/L)	20.13 (13.92,27.28)	20.13 (13.52,25.81)	20.13 (14.00,27.65)	-0.377	0.706
eGFR	6.70 (5.10,8.77)	7.30 (5.35, 9.03)	6.24 (4.75, 8.50)	-1.953	0.051
K (mmol/L)	4.33 (3.88,4.97)	4.38 (3.87,5.00)	4.33 (3.89,4.92)	-0.449	0.654
Na (mmol/L)	140.30 (138.00,142.30)	140.40 (138.00,142.20)	140.18 (138.00,142.70)	-0.186	0.852
Ga (mmol/L)	2.01 (1.87,2.16)	2.01 (1.86,2.16)	2.01 (1.89,2.16)	-0.450	0.653
P (mmol/L)	1.68 (1.37, 2.05)	1.66 (1.29,2.03)	1.71 (1.43, 2.13)	-1.292	0.196
BG (mmol/L)	6.00 (4.92, 7.52)	6.00 (4.94, 7.57)	6.00 (4.91, 7.52)	-0.330	0.742

BI, break-in period; ESRD, end stage renal disease; CGN, chronic glomerulonephritis; PKD, polycystic kidney; WBC, white blood cells; Hb, hemoglobin; Alb, blood albumin; TG, triglyceride; TC, total cholesterol; HDL, high-density lipoprotein cholesterol; LDL, low-density lipoprotein cholesterol; Cr, creatinine; UA, blood uric acid; BUN, blood urea nitrogen; eGFR, estimated glomerular filtration rate; K, blood potassium; Na, blood sodium; Ca, blood calcium; P, blood phosphorus; BG, blood glucose.

### Mechanical Complications

Mechanical complications that occurred in the first 6 months after catheter insertion are presented in **Table 2**. At each follow-up time point, no significant between-group differences in the occurrence of mechanical complications were observed (*p*>0.05) (**Table 2**). The percentage of patients who experienced catheter leakage, bleeding, catheter migration, and omental wrap within 6 months in the BI ≤24 h and BI >24 h groups were 3.2% and 2.6%, 0% and 2.6%, 3.9% and 4.5%, and 1.3% and 0.6%, respectively.

**Table 2:** Mechanical complications between different BI in diabetics with urgent PD within varies follow-up time.

	≤ 24 h (n=155)	> 24 h (n=155)	*p-*value
**Within 2 weeks [n (%)]**			
Leakage	4 (2.6%)	4 (2.6%)	1.000
Bleeding	0 (0.0%)	4 (2.6%)	0.131
Migration	6 (3.9%)	5 (3.2%)	0.759
Omental wrap	0 (0.0%)	1 (0.6%)	1.000
**Within 1 month [n (%)]**			
Leakage	4 (2.6%)	4 (2.6%)	1.000
Bleeding,	0 (0.0%)	4 (2.6%)	0.131
Migration	6 (3.9%)	7 (4.5%)	0.777
Omental wrap	1 (0.6%)	1 (0.6%)	1.000
**Within 3 months [n (%)]**			
Leakage	4 (2.6%)	4 (2.6%)	1.000
Bleeding	0 (0.0%)	4 (2.6%)	0.131
Migration	6 (3.9%)	7 (4.5%)	1.000
Omental wrap	2 (1.3%)	1 (0.6%)	1.000
**Within 6 months [n (%)]**			
Leakage	5 (3.2%)	4 (2.6%)	1.000
Bleeding	0 (0.0%)	4 (2.6%)	0.131
Migration	6 (3.9%)	7 (4.5%)	0.777
Omental wrap	2 (1.3%)	1 (0.6%)	1.000

BI, break-in period; PD, peritoneal dialysis.

**Figure 2:**
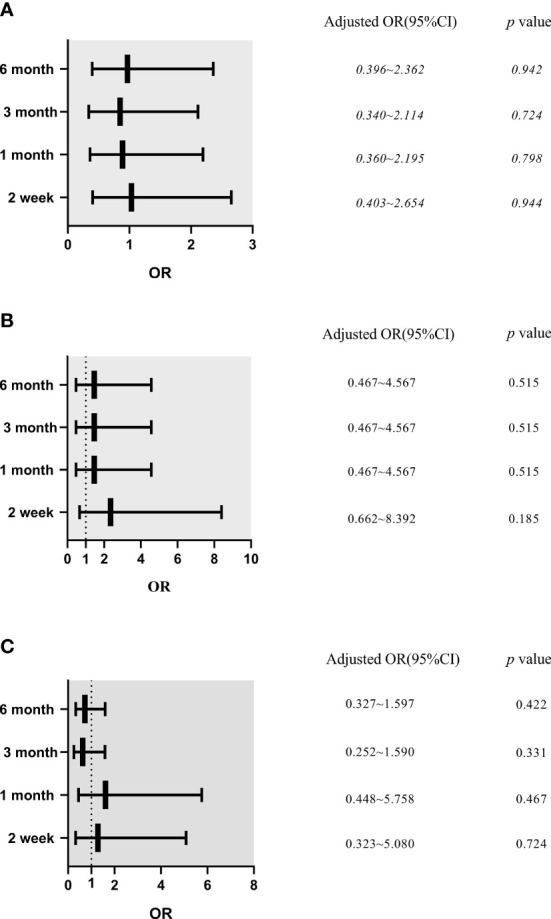
The effects of break-in period on mechanical complications, catheter migration and infectious complications in different follow-up time (Logistic Multivariate Analysis). **(A)** Mechanical complications. Model was adjusted for peritoneal dialysis center, age, temporary hemodialysis usage, abdominal surgery history, white blood cells, hemoglobin, creatinine, blood urea nitrogen, blood potassium, blood phosphorus. **(B)** Catheter migration. Model was adjusted for peritoneal dialysis center, temporary hemodialysis usage, hypertension, abdominal surgery history, white blood cells, blood urea nitrogen and blood phosphorus. **(C)** Infectious complications. Model was adjusted for peritoneal dialysis center, temporary hemodialysis usage, sex, cause of end stage renal disease, hypertension, abdominal surgery history, white blood cells, hemoglobin, blood albumin, high-density lipoprotein cholesterol, low-density lipoprotein cholesterol, blood urea nitrogen, blood uric acid, creatinine, blood calcium, blood phosphorus and blood glucose. OR, odds ratio; CI, confidence interval.

Results of multiple logistic regression analysis showed that a BI ≤24 h was not an independent risk factor for mechanical complications after adjustment for PD center, age, temporary HD usage, and a history of abdominal surgery, as well as levels of WBC, Hb, Cr, BUN, K, and P (*p*>0.05) (**Figure 2A**). Similarly, after adjusting for PD center, temporary HD usage, hypertension, and a history of abdominal surgery, as well as levels of WBC, BUN and P, a BI ≤24 h was not found to be an independent risk factor for catheter migration (*p*>0.05) (**Figure 2B**).

### Infectious Complications

The percentage of patients diagnosed with a tunnel infection within 6 months in the BI ≤24 h versus BI >24 h groups were 0.6% versus 0%, respectively, whereas the percentage of patients diagnosed with peritonitis in the BI ≤24 h versus BI >24 h groups were 12.3% versus 14.2%, respectively. At each time point, there was no between-group difference in the occurrence of infectious complications. Peritonitis was the most common infectious complication (**Table 3**).

**Table 3:** Infectious complications between different BI in diabetics with urgent PD within varies follow-up time.

	≤ 24 h (n=155)	> 24 h (n=155)	*p*-value
**Within 2 weeks [n (%)]**			
Tunnel infection	1 (0.6%)	0 (0.0%)	0.317
Peritonitis	5 (3.2%)	5 (3.2%)	1.000
**Within 1 month [n (%)]**			
Tunnel infection	1 (0.6%)	0 (0.0%)	0.317
peritonitis	8 (5.2%)	7 (4.5%)	0.791
**Within 3 months [n (%)]**			
Tunnel infection	1 (0.6%)	0 (0.0%)	0.317
peritonitis	14 (9.0%)	16 (10.3%)	0.701
**Within 6 months [n (%)]**			
Tunnel infection	1 (0.6%)	0 (0.0%)	0.317
peritonitis	19 (12.3%)	22 (14.2%)	0.615

BI, break-in period; PD, peritoneal dialysis.

After adjusting for PD center, temporary HD usage, sex, cause of ESRD, hypertension, and a history of abdominal surgery, as well as levels of WBC, Hb, Alb, HDL, LDL, BUN, UA, Cr, Ca, P, and BG, a BI ≤24 h could not be considered as an independent risk factor for infectious complications (*p*>0.05) (**Figure 2C**).

### Technique Survival

**Figure 3:**
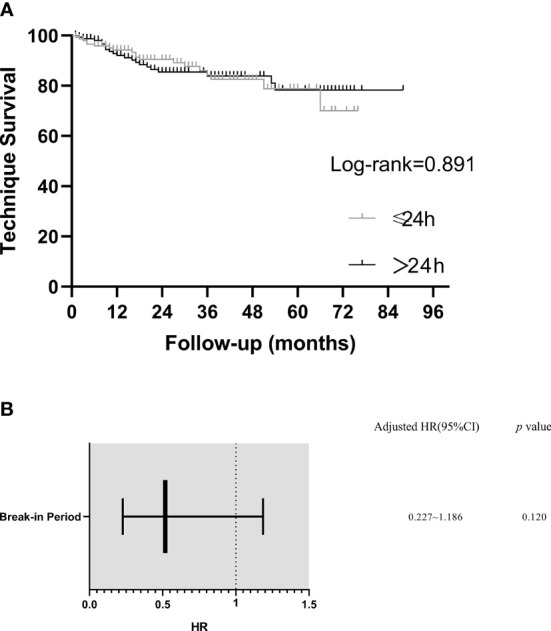
Kaplan–Meier plot of technique survival analysis in different break-in period group and predictors of technique failure (Cox Multivariate Analysis). **(A)** Technique survival curves. **(B)** Predictors of technique failure. Model was adjusted for peritoneal dialysis center, age, hypertension, white blood cells, blood urea nitrogen and blood potassium. HR, hazard ratio; CI, confidence interval.

After 1, 2, and 3 years, technique survival rates were 94.4% and 91.8%, 89.8% and 85.9%, and 83.8% and 84.3% in the BI ≤24 h and BI >24 h groups, respectively. No significant between-group difference in technique survival rate was demonstrated (log-rank: *p*=0.891) (**Figure 3A**).

In a multivariable Cox analysis including PD center, age, hypertension, WBC, BUN and K in the model, BI ≤ 24h was not an independent predictor for technique failure (HR= 0.518, 95%CI =0.227–1.186, *p* > 0.05) (**Figure 3B**).

## Discussion

To the best of our knowledge, this is the first study that focuses on the feasibility of applying a BI ≤24 h in patients with diabetes undergoing USPD. We found that, similar to patients who underwent PD initiation >24 h after catheter insertion, those who underwent PD initiation ≤24 h after catheter insertion did not have a higher risk of complications and had comparable technique survival rates.

It is particularly important to monitor the quality of catheter implantation procedures in different PD centers. In our research, professionally trained and experienced doctors performed the surgery. Additionally, the variable “PD center” was corrected for in our analysis to reduce the effect of different dialysis centers on the results. In addition, the patient’s preoperative nutritional status is also an important factor affecting short-term postoperative complications. Baseline data of patients in this study showed that Hb, Alb and other indicators were comparable between the two groups. Similarly, we corrected for the above nutritional indicators in multivariate regression analysis, thus avoiding their influence on the results.

Many factors may contribute to mechanical complications in patients undergoing PD. For instance, the method of catheter implantation surgery, the initial dialysate volume, a history of abdominal surgery, and the BI can affect the incidence of mechanical complications (5, 9, 23). As previously suggested, a shorter BI is associated with a higher occurrence of mechanical complications in general patients undergoing PD (12, 13). Liu et al. (12) and Kim et al. (13) investigated the feasibility of USPD with a BI ≤7 days and BI ≤48 h, respectively. Both studies concluded that patients who underwent USPD presented a much higher risk of early mechanical complications, such as catheter malposition, in the shorter BI than in the longer BI group. Liu et al. explained that a shorter BI could lead to catheter floating and increased pressure in the peritoneal cavity, which might lead to catheter leakage and malposition (12). Unfortunately, the two aforementioned studies did not directly compare the complications in patients with diabetes. Ranganathan et al. (24) in Australia evaluated 122 patients who underwent PD, including 43 patients with diabetes. Patients who underwent PD initiation at 1, 2, and 4 weeks after the insertion of a PD catheter were assigned to groups 1, 2, and 3, respectively. Among the patients with diabetes, the incidence of catheter leakage in group 1 (46.7%) was significantly higher than that in groups 2 (14.3%) and 3 (7.1%). A shorter BI delays wound healing, which may increase the risk of catheter leakage (12). Furthermore, wound healing is more complicated in patients with diabetes (16). However, we found that patients with diabetes who underwent USPD initiation with a BI ≤24 h did not have an increased incidence of mechanical complications. Additionally, our study showed that the BI was not an independent risk factor for mechanical complication. In our study, the most common short-term mechanical complication among patients who underwent USPD was catheter tip migration, which corroborated with a previous study finding (13). We also found that the BI was not an independent risk for catheter migration. This finding may be related to the following reasons: First, open surgery has the advantage of direct visualization, which may reduce the risk of catheter malposition. Second, purse-string sutures were used to reduce the risk of leakage. Finally, a low initial dwell volume could reduce abdominal pressure, and thus reduce the incidence of catheter leakage.

Infection is another common complication in patients undergoing PD. Peritonitis associated with PD is the main reason for hospital admission and referral for HD (25, 26). In the literature, the proportion of patients diagnosed with peritonitis after USPD reportedly varies from 4.0% to 15.4% during the 6-month follow-up period (1, 7, 12, 13). Reduced residual renal function, low Alb and catheter leakage are considered risk factors for peritonitis (27, 28). Liu et al. and Kim et al. evaluated the relationship between the BI and incidence of infectious complications in patients undergoing PD; they found that the BI did not affect the incidence of infectious complication. Moreover, Ranganathan et al. (24) performed a subgroup analysis of ESRD patients with diabetes. In their experience, the proportions of infectious complications in groups with BIs of 1, 2, and 4 weeks were 13.3%, 0.0%, and 7.1%, respectively, over a 4-week follow-up period. The three treatment groups showed no difference in the infection risk. Consistent with the aforementioned studies, we found no difference in the incidence of infectious complications within 6 months between patients in the different BI groups. It is well established that PD center factors affect the risk of peritonitis (29). In multiple logistic regression analysis, we adjusted for the PD center as a covariate and determined that a BI ≤24 h was not a significant risk factor for infectious complications. For USPD patients, avoiding catheter leakage, prophylactic antibiotic administration, aseptic processing procedures, and operational process education are beneficial to reduce infection (28, 30).

It has been suggested that USPD may have no important implications on technique survival (12, 13). Jin et al. (31) evaluated 50 patients with diabetes who underwent USPD with a BI ≤14 days and reported that the technique survival rates at 12 and 36 months were 98.0% and 93.7%, respectively. Unfortunately, no subgroup analyses were performed in terms of the BI. In the current study, we observed technique survival rates that were similar to those reported by Jin et al. (31). Importantly, we found that a BI ≤24 h was not an independent risk factor for technique failure, probably because catheter insertion was performed by a seasoned nephrologist using open surgery, strong purse-string sutures, a low initial dwell volume, and prophylactic antibiotic administration perioperatively, which considerably lowered early technique failure resulting from catheter-related complications. Our patient population had a low incidence of mechanical and infectious complications. Therefore, a shorter BI did not affect technique failure.

We believe that USPD is an alternative treatment modality for late-presenting patients with ESRD and diabetes. A previous small sample, single-center study confirmed the feasibility of initiating dialysis within 14 days in ESRD patients with diabetes (31); however, our study found that it is also safe to initiate dialysis within 24 h after catheter insertion. The advantages of this retrospective study could be summarized as follows: First, we included the largest cohort of patients with diabetes who underwent USPD. Although different catheterization methods may have affected the incidence of PD-related complications (14), all patients were treated with open surgery. Second, in the multivariate analysis, we considered most of the recognized confounders simultaneously. Third, we included patients undergoing temporary HD, who usually have poor initial clinical statuses; hence, our study was different from previous studies (1, 31, 32) and was more reflective of real-world situations. Generally, patients undergoing temporary HD have worse baseline conditions than those not undergoing temporary HD, which may affect the incidence of short-term complications in patients undergoing USPD. By correcting for the variable “temporary HD usage” in our analysis, we found that a BI ≤24 h still did not affect the occurrence of short-term mechanical and infectious complications in patients with diabetes undergoing USPD, thereby indicating that the results of this study are widely applicable.

Nevertheless, our research has several limitations. First, some data were not recorded in this study, such as the levels of brain natriuretic peptide and cardiac markers. Therefore, it was not possible to perform a risk factor analysis of these parameters. Second, our research was conducted in Northeast China, and thus our findings may not be generalized to other parts of the world. Lastly, the generalizability of our study findings is limited by the non-randomized and retrospective design. Future prospective, randomized controlled trials are required to confirm the optimal timing of USPD initiation in patients with diabetes.

## Conclusion

For most ESRD patients with diabetes, it may be feasible to commence dialysis immediately (BI ≤24 h) after catheter implantation.

## Data Availability Statement

The datasets used and analyzed during the current study are available from the corresponding author on reasonable request.

## Ethics Statement

Informed consent from the subjects was waived due to the retrospective aspect of the study. This study was approved by the Ethics Committee of The Second Hospital of Jilin University (No. 2020031, retrospectively registered). The research was conducted in compliance with the Declaration of Helsinki.

## Author Contributions

WC designed this study. LY, ZS, XZ, XZ provided data. XH, XW, SL collected data. XH analyzed data and wrote the manuscript. WC and WZ reviewed the manuscript. All the authors read and approved the final manuscript.

## Funding

This study was supported by Jilin Province Health and Technology Innovation Development Program Funded Project, 2019SRCJ015.

## Conflict of Interest

The authors declare that the research was conducted in the absence of any commercial or financial relationships that could be construed as a potential conflict of interest.

## Publisher’s Note

All claims expressed in this article are solely those of the authors and do not necessarily represent those of their affiliated organizations, or those of the publisher, the editors and the reviewers. Any product that may be evaluated in this article, or claim that may be made by its manufacturer, is not guaranteed or endorsed by the publisher.
